# Comparative analysis of volatile organic compounds in different parts of *Poria cocos*

**DOI:** 10.3389/fchem.2026.1777381

**Published:** 2026-04-01

**Authors:** Qiuye Liu, Haili Tang, Ping Xie, Junmei Huang, Yajie Zuo, Min Wen

**Affiliations:** 1 The First Hospital of Hunan University of Chinese Medicine, Hunan University of Chinese Medicine, Changsha, China; 2 Academy of Chinese Medical Sciences, Hunan University of Chinese Medicine, Changsha, China

**Keywords:** GC-IMS, Poria, Poria cum radice pini, Poriae cutis, volatile organic compounds

## Abstract

**Introduction:**

*Poria cocos*, a fungus recognized for both its edible and medicinal properties, is highly valued for its bioactive effects, such as immune modulation, improvement of digestive function, inflammation reduction, and enhancement of sleep quality. Typically, three parts of Poria cocos --Poria, Poriae cutis, and Poria cum radice pini --are utilized in functional foods.

**Methods:**

This research applied Gas Chromatography -Ion Mobility Spectrometry (GC-IMS) alongside chemometric techniques to compare the volatile organic compounds (VOCs) present in these three sections. Analytical methods including Principal Component Analysis (PCA), Cluster Analysis (CA), Euclidean Distance Analysis, and Partial Least-Squares Discriminant Analysis (PLS-DA) were used.

**Results:**

The study identified 104 VOCs, predominantly aldehydes, ketones, alcohols, and terpenoids. Among them, PC-01 and PC-03 contain two compounds that PC-02 lacks, and PC-02 and PC-03 contain ten compounds that PC-01 lacks. Furthermore, 3-octanone and 4-methyl-3-penten-2-one are unique to PC-02, while β-Ocimene, 1,8-cineole, 2-methyl-2-pentenal, and Sabine are unique to PC-03. The findings lay the groundwork for the precise application of specific parts of *Poria cocos* in dietary and medicinal contexts.

**Conclusion:**

In particular, the study helps to clarify the biochemical factors contributing to the anxiolytic effects of Poria cum radice pini, such as L-Perillaldehyde, which is a neuroactive compound, and linalool, which is recognized for its anti-anxiety, anti-tumor, sedative, and hypnotic properties. This study thereby supports targeted innovations in the development of health products from various parts of *Poria cocos*.

## 1 Introduction

*Poria cocos* is a functional fungus known for its culinary and medicinal applications; it is predominantly found in Asia, the Americas, Oceania, and parts of Africa, with China being the leading cultivator (Lei et al., 2025). Because it is safe to eat, it is often incorporated into a range of food products—including dietary dishes, congees, pastries, and other healthful options—which underscores its value as an edible resource (Gao et al., 2024a). Its therapeutic benefits are largely attributed to active compounds such as polysaccharides, terpenoids (especially triterpenoids), and volatile organic compounds (VOCs) (Gao et al., 2024b). Additionally, *P. cocos* is esteemed for its broad spectrum of pharmacological actions, which include immunomodulatory effects (Bai et al., 2024; Sun et al., 2024; Wei et al., 2025), the alleviation of functional dyspepsia (Tu et al., 2022), anti-inflammatory properties (Bao et al., 2022), protection against alcoholic liver injury (Jiang et al., 2022), enhancement of sleep quality (Kim et al., 2022), and antioxidant activities (Gao et al., 2024a; Gao et al., 2024b), all of which contribute to its growing popularity among consumers.

The nutritional and therapeutic attributes of *P. cocos* vary considerably among its different parts. Specifically, Poria—the dried sclerotium—is commonly utilized in traditional medicine to address conditions related to spleen deficiency, such as diarrhea and indigestion (Zou et al., 2020). Poriae cutis, which is the dried outer layer of the sclerotium, is typically employed to promote diuresis and exert anti-inflammatory effects (Jin et al., 2024). In contrast, Poria cum radice pini, the section of the sclerotium that naturally encloses a pine root, has been shown to have sedative, tranquilizing, and sleep-improving properties (Zhang et al., 2009). Research has revealed significant differences among these parts in terms of their polysaccharide and triterpenoid contents (Zhang et al., 2019; Zhu et al., 2018; Zhu et al., 2019); for instance, Poria tends to contain higher levels of polysaccharides (Song et al., 2015), whereas Poriae cutis is richer in triterpenoid acids such as poricoic acid and pachymic acid (Zhan et al., 2023). At present, research on Poria cocos mainly focuses on cultivation substrates, processing methods, or drying techniques, while there is little research on the VOCs of different medicinal parts of Poria cocos.

While present in relatively small quantities, the VOCs in *P. cocos* are notably diverse and contribute significantly to both its pharmacological properties and sensory profile. For example, terpenoids and various aromatic components exhibit a spectrum of bioactivities, including sedative, antidepressant, antimicrobial, antiviral, anti-inflammatory, and antioxidant effects. Additionally, these volatile compounds are chiefly responsible for the unique aroma of *P. cocos*, which not only activates the olfactory system and stimulates appetite but also enhances its overall taste and flavor (Liao et al., 2008; Zhang et al., 2009). Moreover, in advanced processing applications, these compounds can be leveraged as natural additives to improve the quality and market value of food products.

While conventional techniques such as Headspace Gas Chromatography-Mass Spectrometry (HS-GC-MS), Headspace Solid-Phase Microextraction Gas Chromatography-Mass Spectrometry (HS-SPME-GC-MS), and Comprehensive Two-Dimensional Gas Chromatography-Mass Spectrometry (GC × GC-MS) are widely used for VOC analysis, they typically involve longer sample preparation and analysis times. In contrast, gas chromatography–ion mobility spectrometry (GC-IMS) enables rapid VOC profiling with minimal sample pretreatment, while offering high sensitivity and intuitive visual data interpretation. Unlike MS-based methods, GC-IMS generates intuitive fingerprint spectra that allow direct visual comparison of sample differences without complex data processing. These features make GC-IMS particularly suitable for high-throughput screening and routine quality control in food research (Ai et al., 2025; He et al., 2023; Ouyang et al., 2025). Owing to these strengths, GC-IMS has established itself as an essential method within the food industry. It plays a critical role in flavor profiling, quality evaluation, and process monitoring by capitalizing on its exceptional sensitivity, quick detection speed, and effective visual data analysis.

This research utilizes GC-IMS to compare the VOCs present in three parts of Poria cocos. To interpret the VOC data, multiple chemometric methods were employed: Principal Component Analysis (PCA) for visualizing clustering trends, Cluster Analysis (CA) for validating grouping patterns, Partial Least-Squares Discriminant Analysis (PLS-DA) for identifying discriminating compounds, and Euclidean distance for quantifying inter-group dissimilarities. This complementary approach ensures robust interpretation of the volatile profiles. This study is expected to broaden the applications of Poria cocos for both medicinal and culinary purposes, enhance its overall value, and support a deeper investigation into its active components.

## 2 Materials and methods

### 2.1 Materials

Fresh *P. cocos* was collected in Chu Xiong, Yunnan Province, China, on 24 February 2025, a sunny day with a temperature of 25 °C and a relative humidity of 55%. The surrounding area is well drained soil and biological communities such as *Pinus yunnanensis*.

**FIGURE 1:**
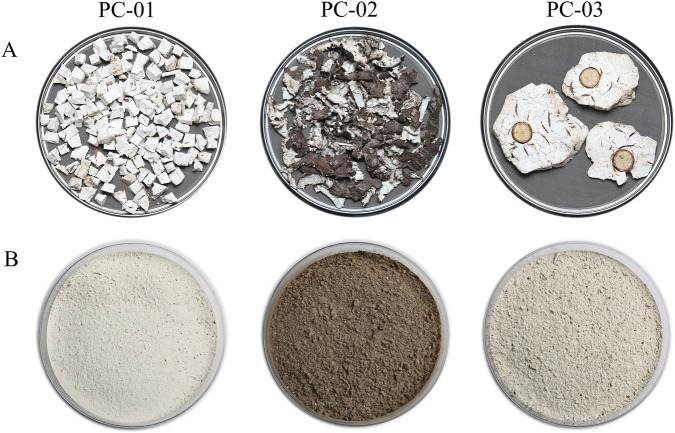
Photos of different parts of *Poria cocos* **(A)** and powders **(B)**. PC-01: Poria; PC-02: Poriae cutis; PC-03: Poria cum radius pini.

After rinsing to eliminate surface impurities, the outer layer of the fresh sclerotium was manually removed. The inner white tissue was then diced into cubes ranging from 0.5 to 2.0 cm per side and labeled as Poria (PC-01). The detached skin was designated as Poriae cutis (PC-02), and the part of the sclerotium naturally surrounding a pine root was identified as Poria cum radice pini (PC-03). Each sample was subsequently dried in an oven at 60 °C for 6 h, ground into a fine powder, and stored for later analysis. Three samples were analyzed, including Poria (PC-01), Poriae cutis (PC-02), and Poria cum radius pini (PC-03), and each sample was analyzed in triplicate. Figure 1 provides a schematic diagram of the various parts of the *P. cocos*.

### 2.2 Analysis of VOCs by GC-IMS

Based on the method outlined by He et al. (2023) with some modifications.

The analysis utilized a FlavourSpec® gas chromatography–ion mobility spectrometry (GC-IMS) from G.A.S. (Dortmund, Germany), paired with a CTC-PAL 3 static headspace autosampler from CTC Analytics AG (Zwingen, Switzerland). Data were acquired and processed using VOCal software (version 0.4.03) supplied by G.A.S. (Dortmund, Germany). For the separation step, an MXT-WAX capillary column (30 m × 0.53 mm, 1.0 μm) from Restek Corporation (United States) was employed.

In brief, 1 g of the sample was measured and placed into a 20 mL headspace vial. The vial was then incubated at 80 °C for 20 min before injection. Injected at a constant and slow rate, and the temperature is 85 °C.

The headspace injection was performed under the following conditions: the incubation temperature was set at 80 °C for 20 min, with an injection volume of 500 µL using the splitless mode, an agitation speed during incubation of 500 r/min, and a syringe temperature maintained at 85 °C.

The gas chromatography analysis was conducted with the column temperature held at 60 °C, using high-purity nitrogen (N_2_) (≥99.999%) as the carrier gas. The flow rate was programmed to start at 2.0 mL/min for the first 2 min, and it was then linearly increased to 10.0 mL/min over the next 8 min, followed by a further linear ramp to 100.0 mL/min over an additional 10 min. This rate was maintained at 100.0 mL/min for the final 40 min, resulting in a total run time of 60 min. The injector port was maintained at 80 °C throughout the procedure.

IMS conditions: A tritium (^3^H) radioactive source served as the ionization mechanism. The migration tube, with a length of 53 mm, operated under an electric field strength of 500 V/cm and was held at a constant temperature of 45 °C. Nitrogen (N_2_) with a minimum purity of 99.999% was employed as the drift gas at a flow rate of 75.0 mL/min. All experiments were carried out in positive ion mode.

### 2.3 Statistical analysis

A mixture of six ketones (2-butanone, 2-pentanone, 2-hexanone, 2-heptanone, 2-octanone, and 2-nonanone) (Each is 10 ppm, Analytical Reagent) was used to construct calibration curves for both the retention time and the retention index (Supplementary Figure S1). Each compound’s retention index was then determined based on its measured retention time. For qualitative identification, the observed retention indices and drift times were compared with the GC retention index database (NIST, 2020) and the IMS drift time database provided within the VOCal software (version 2.0.0, G.A.S., Dortmund, Germany). Using the Reporter, Gallery Plot, and Dynamic PCA plugins in VOCal, comprehensive data—including three-dimensional spectra, two-dimensional spectra, differential spectra, fingerprint maps, and PCA score plots—were generated for a comparative analysis of the volatile organic compounds across samples. Furthermore, cluster analysis (CA) and partial least-squares regression analysis (PLS-DA) were carried out using TBtools and SIMCA (Version 14.1, Umetrics, Sweden), respectively. Venn diagram was drawn using OriginPro 2026 (OriginLab Co., Northampton, MA, United States).

## 3 Results

### 3.1 Analysis of GC-IMS results

#### 3.1.1 Comparative analysis of VOCs in three parts of the *Poria cocos* samples

**FIGURE 2:**
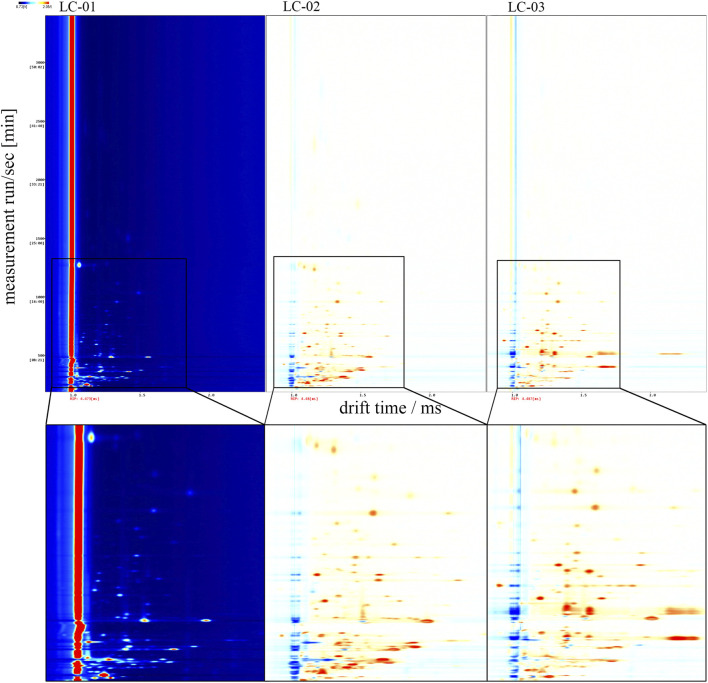
Differential spectrum of VOCs in three parts of the *Poria cocos* samples. PC-01: Poria; PC-02: Poriae cutis; PC-03: Poria cum radius pini.

Figure 2 shows the differential comparison plots created by subtracting the spectrum of a designated *Poria cocos* sample (used as the reference) from those of the other samples. When a volatile organic compound is present in equal amounts in the target sample and the reference, the subtraction results in a white background. In contrast, red areas denote that the target sample has a higher level of the compound compared to the reference, while blue areas indicate a lower level. These plots effectively highlight the differences in volatile components across various parts of *P. cocos*.

GC-IMS detected a total of 118 compounds. By comparing the data with the GC retention index (NIST 2020) and IMS drift time databases, 104 VOCs were identified across the three group samples, 14 compounds have not been identified yet. In total, 29 Aldehydes, 20 Alcohols, 17 Ketones, 14 Terpenes, 7 Nitrogen-containing heterocycles, 5 Esters, 4 Acids, 4 Furans, 3 Sulfur-containing compounds, 1 Aromatic hydrocarbons were identified (Table 1).

**TABLE 1:** Detail of the VOCs in three parts of the Poria cocos samples.

Compound category	No	Compound	CAS	Molecular formula	MW	RI	Rt (s)	Dt (ms)
Aldehydes	1	(E)-2-Butenal	C123739	C_4_H_6_O	70.1	1070.0	435.617	1.19901
	2	(E)-2-Heptenal-M	C18829555	C_7_H_12_O	112.2	1337.0	880.857	1.26115
		(E)-2-Heptenal-D	C18829555	C_7_H_12_O	112.2	1337.0	880.857	1.66593
	3	(E)-2-Hexenal-M	C6728263	C_6_H_10_O	98.1	1237.0	714.782	1.18542
		(E)-2-Hexenal-D	C6728263	C_6_H_10_O	98.1	1237.0	714.782	1.52361
	4	(E)-2-Nonenal	C18829566	C_9_H_16_O	140.2	1581.3	1493.034	1.41631
	5	(E)-2-Octenal-M	C2548870	C_8_H_14_O	126.2	1445.9	1114.386	1.33810
		(E)-2-Octenal-D	C2548870	C_8_H_14_O	126.2	1445.9	1114.386	1.81496
	6	(E)-2-Pentenal-M	C1576870	C_5_H_8_O	84.1	1154.2	572.887	1.10720
		(E)-2-Pentenal-D	C1576870	C_5_H_8_O	84.1	1154.2	572.887	1.35968
	7	(E)-2-Undecenal	C53448070	C_11_H_20_O	168.3	1885.2	2879.030	1.54696
	8	(E,E)-2,4-heptadienal	C4313035	C_7_H_10_O	110.2	1522.8	1315.934	1.20364
	9	2,6-Nonadienal	C557482	C_9_H_14_O	138.2	1701.2	1934.943	1.36867
	10	2-Ethylbutanal	C97961	C_6_H_12_O	100.2	1017.9	374.867	1.50666
	11	2-Methyl-2-pentenal	C623369	C_6_H_10_O	98.1	1171.9	608.772	1.15948
	12	2-Methyl-2-propenal	C78853	C_4_H_6_O	70.1	900.9	285.951	1.22016
	13	2-Methylpropanal	C78842	C_4_H_8_O	72.1	830.1	244.990	1.28227
	14	2-Propenal	C107028	C_3_H_4_O	56.1	869.5	266.969	1.05888
	15	3-Methyl-2-butenal-M	C107868	C_5_H_8_O	84.1	1219.7	689.949	1.09444
		3-Methyl-2-butenal-D	C107868	C_5_H_8_O	84.1	1219.7	689.949	1.36095
	16	3-Methylbutanal	C590863	C_5_H_10_O	86.1	934.4	307.597	1.40047
	17	Acetaldehyde	C75070	C_2_H_4_O	44.1	770.4	215.019	1.03484
	18	Benzaldehyde	C100527	C_7_H_6_O	106.1	1555.4	1411.989	1.15806
	19	Butanal	C123728	C_4_H_8_O	72.1	898.8	284.619	1.28127
	20	Decanal	C112312	C_10_H_20_O	156.3	1537.4	1357.958	1.55580
	21	Furfural	C98011	C_5_H_4_O_2_	96.1	1501.8	1257.547	1.08832
	22	Heptanal-M	C111717	C_7_H_14_O	114.2	1201.8	665.213	1.35585
		Heptanal-D	C111717	C_7_H_14_O	114.2	1201.8	665.213	1.69505
	23	Hexanal-M	C66251	C_6_H_12_O	100.2	1103.8	481.954	1.28317
		Hexanal-D	C66251	C_6_H_12_O	100.2	1104.2	482.651	1.56498
	24	L-perillaldehyde	C18031408	C_10_H_14_O	150.2	1926.2	3146.270	1.30395
	25	Nonanal-M	C124196	C_9_H_18_O	142.2	1410.1	1031.394	1.49038
		Nonanal-D	C124196	C_9_H_18_O	142.2	1410.5	1032.431	1.94186
	26	Octanal-M	C124130	C_8_H_16_O	128.2	1304.6	821.138	1.42176
		Octanal-D	C124130	C_8_H_16_O	128.2	1304.9	821.707	1.82392
	27	Pentanal-M	C110623	C_5_H_10_O	86.1	1006.3	362.545	1.20814
		Pentanal-D	C110623	C_5_H_10_O	86.1	1006.6	362.878	1.42351
	28	Phenylacetaldehyde	C122781	C_8_H_8_O	120.2	1783.9	2313.162	1.24775
	29	Propanal	C123386	C_3_H_6_O	58.1	821.3	240.328	1.14303
Alcohols	30	1-Butanol-M	C71363	C_4_H_10_O	74.1	1165.2	594.836	1.18753
		1-Butanol-D	C71363	C_4_H_10_O	74.1	1165.3	595.184	1.38901
	31	1-Heptanol	C111706	C_7_H_16_O	116.2	1494.5	1237.837	1.41023
	32	1-Hexanol-M	C111273	C_6_H_14_O	102.2	1375.8	957.739	1.33142
		1-Hexanol-D	C111273	C_6_H_14_O	102.2	1375.8	957.739	1.64132
	33	1-Octanol	C111875	C_8_H_18_O	130.2	1662.1	1777.995	1.48063
	34	1-Octen-3-ol	C3391864	C_8_H_16_O	128.2	1491.8	1230.575	1.16445
	35	1-Pentanol-M	C71410	C_5_H_12_O	88.1	1270.6	765.401	1.25985
		1-Pentanol-D	C71410	C_5_H_12_O	88.1	1270.6	765.401	1.51577
	36	1-Penten-3-ol-M	C616251	C_5_H_10_O	86.1	1180.5	626.889	0.94015
		1-Penten-3-ol-D	C616251	C_5_H_10_O	86.1	1180.3	626.540	1.47954
	37	1-Propanol-M	C71238	C_3_H_8_O	60.1	1059.9	423.075	1.11357
		1-Propanol-D	C71238	C_3_H_8_O	60.1	1060.7	424.153	1.25522
	38	2-Ethyl-1-hexanol	C104767	C_8_H_18_O	130.2	1545.5	1381.971	1.42598
	39	2-Methyl-1-propanol	C78831	C_4_H_10_O	74.1	1115.1	501.116	1.17350
	40	2-Methyl-2-propanol	C75650	C_4_H_10_O	74.1	938.8	310.594	1.33135
	41	2-Propanol	C67630	C_3_H_8_O	60.1	940.8	311.927	1.09394
	42	3-Methyl-1-butanol-M	C123513	C_5_H_12_O	88.1	1226.0	698.857	1.24810
		3-Methyl-1-butanol-D	C123513	C_5_H_12_O	88.1	1226.3	699.356	1.49230
	43	3-Methyl-2-butanol	C598754	C_5_H_12_O	88.1	1113.3	497.981	1.42981
	44	α-Terpineol	C98555	C_10_H_18_O	154.3	1798.6	2387.776	1.22237
	45	Borneol	C507700	C_10_H_18_O	154.3	1863.4	2746.734	1.22063
	46	cis-2-Penten-1-ol	C1576950	C_5_H_10_O	86.1	1342.7	891.663	0.94386
	47	Ethanol-M	C64175	C_2_H_6_O	46.1	950.4	318.587	1.04185
		Ethanol-D	C64175	C_2_H_6_O	46.1	950.0	318.254	1.12700
	48	Linalool	C78706	C_10_H_18_O	154.3	1642.4	1704.075	1.22385
	49	(Z)-3-Hexenol	C928961	C_6_H_12_O	100.2	1407.7	1026.207	1.24727
Ketones	50	1-Hydroxy-2-propanone	C116096	C_3_H_6_O_2_	74.1	1319.7	848.438	1.08357
	51	1-Octen-3-one-M	C4312996	C_8_H_14_O	126.2	1316.9	843.320	1.27943
		1-Octen-3-one-D	C4312996	C_8_H_14_O	126.2	1317.2	843.888	1.67768
	52	1-Penten-3-one	C1629589	C_5_H_8_O	84.1	1049.4	410.499	1.07791
	53	2,3-Butanedione	C431038	C_4_H_6_O_2_	86.1	1002.5	358.549	1.18209
	54	2-Butanone	C78933	C_4_H_8_O	72.1	920.8	298.606	1.24420
	55	2-Heptanone	C110430	C_7_H_14_O	114.2	1198.7	661.032	1.26149
	56	2-Pentanone	C107879	C_5_H_10_O	86.1	1005.4	361.546	1.35139
	57	3-Hydroxy-2-butanone	C513860	C_4_H_8_O_2_	88.1	1303.9	820.001	1.08096
	58	3-Methyl-2-cyclopenten-1-one	C2758181	C_6_H_8_O	96.1	1577.5	1481.028	1.09454
	59	3-Methyl-2-pentanone	C565617	C_6_H_12_O	100.2	1035.0	393.849	1.50265
	60	3-Octanone-M	C106683	C_8_H_16_O	128.2	1269.9	764.263	1.31208
		3-Octanone-D	C106683	C_8_H_16_O	128.2	1269.9	764.263	1.71424
	61	4-Methyl-3-penten-2-one-M	C141797	C_6_H_10_O	98.1	1155.6	575.674	1.12887
		4-Methyl-3-penten-2-one-D	C141797	C_6_H_10_O	98.1	1155.4	575.325	1.44639
	62	6-Methyl-5-hepten-2-one	C110930	C_8_H_14_O	126.2	1352.9	911.570	1.18020
	63	Acetone	C67641	C_3_H_6_O	58.1	841.8	251.318	1.11598
	64	Camphor	C76222	C_10_H_16_O	152.2	1598.6	1550.067	1.34312
	65	Carvone	C99490	C_10_H_14_O	150.2	1829.9	2555.016	1.30299
	66	Cyclohexanone	C108941	C_6_H_10_O	98.1	1299.7	812.607	1.15408
Terpenes	67	1,8-Cineole-M	C470826	C_10_H_18_O	154.3	1218.4	688.207	1.30612
		1,8-Cineole-D	C470826	C_10_H_18_O	154.3	1218.4	688.207	1.72055
	68	3-Carene	C13466789	C_10_H_16_	136.2	1145.7	556.512	1.30485
	69	α-Pinene	C80568	C_10_H_16_	136.2	1041.1	400.842	1.21515
	70	α-Terpinene	C99865	C_10_H_16_	136.2	1190.5	648.838	1.21558
	71	β-Ocimene-M	C13877913	C_10_H_16_	136.2	1220.7	691.343	1.21686
		β-Ocimene-D	C13877913	C_10_H_16_	136.2	1221.2	692.039	1.65807
	72	β-Pinene	C127913	C_10_H_16_	136.2	1122.0	512.962	1.21558
	73	Camphene	C79925	C_10_H_16_	136.2	1081.2	449.901	1.20921
	74	γ-Terpinene	C99854	C_10_H_16_	136.2	1259.2	747.770	1.21806
	75	Limonene	C138863	C_10_H_16_	136.2	1211.2	678.103	1.21686
	76	Myrcene	C123353	C_10_H_16_	136.2	1179.7	625.147	1.21558
	77	Sabinene-M	C3387415	C_10_H_16_	136.2	1132.8	532.472	1.21558
		Sabinene-D	C3387415	C_10_H_16_	136.2	1132.8	532.472	1.63384
	78	Terpinolene	C586629	C_10_H_16_	136.2	1294.5	803.507	1.21806
	79	Tetrahydrolinalool	C78693	C_10_H_22_O	158.3	1424.3	1063.553	1.26731
	80	Linalool oxide	C60047178	C_10_H_18_O_2_	170.3	1459.9	1148.620	1.26731
Nitrogen-containing heterocycles	81	2,3,5-Trimethylpyrazine	C14667551	C_7_H_10_N_2_	122.2	1452.3	1129.947	1.17113
	82	2,3-Diethyl-5-methylpyrazine	C18138040	C_9_H_14_N_2_	150.2	1509.0	1277.258	1.26196
	83	2,6-Dimethylpyrazine	C108509	C_6_H_8_N_2_	108.1	1328.9	865.501	1.13319
	84	2-Ethyl-5-methylpyrazine	C13360640	C_7_H_10_N_2_	122.2	1436.7	1092.601	1.17113
	85	2-Isopropyl-3-methoxy pyrazine	C25773404	C_8_H_12_N_2_O	152.2	1442.0	1105.049	1.25395
	86	1-Methyl-2-pyrrolidinone	C872504	C_5_H_9_NO	99.1	1806.0	2426.370	1.10145
	87	Pyrrolidine	C123751	C_4_H_9_N	71.1	1035.6	394.515	1.04786
Esters	88	(Z)-3-Hexen-1-yl acetate	C3681718	C_8_H_14_O_2_	142.2	1327.4	862.657	1.30424
	89	Ethyl acetate	C141786	C_4_H_8_O_2_	88.1	906.8	289.614	1.33436
	90	Ethyl nonanoate	C123295	C_11_H_22_O_2_	186.3	1705.5	1952.953	1.55677
	91	Hexyl propionate	C2445763	C_9_H_18_O_2_	158.2	1346.2	898.488	1.44134
	92	Methyl 3-methylbutanoate	C556241	C_6_H_12_O_2_	116.2	1024.3	381.860	1.19211
Acids	93	2-Methylpropanoic acid	C79312	C_4_H_8_O_2_	88.1	1695.0	1909.214	1.17012
	94	Acetic acid-M	C64197	C_2_H_4_O_2_	60.1	1507.1	1272.071	1.05893
		Acetic acid-D	C64197	C_2_H_4_O_2_	60.1	1507.1	1272.071	1.16445
	95	Butanoic acid	C107926	C_4_H_8_O_2_	88.1	1779.7	2292.578	1.16714
	96	Propanoic acid	C79094	C_3_H_6_O_2_	74.1	1640.9	1698.234	1.11937
Furans	97	2,5-Dimethylfuran	C625865	C_6_H_8_O	96.1	952.4	319.919	1.37242
	98	2-Pentylfuran	C3777693	C_9_H_14_O	138.2	1249.0	732.414	1.25201
	99	Dihydro-5-methyl-2(3H)-furanone	C108292	C_5_H_8_O_2_	100.1	1740.7	2107.328	1.13430
	100	γ-Butyrolactone	C96480	C_4_H_6_O_2_	86.1	1715.2	1994.120	1.09100
Sulfur-containing compounds	101	2,5-Dimethyl thiophene	C638028	C_6_H_8_S	112.2	1175.6	616.437	1.07659
	102	2-Furylmethanethiol	C98022	C_5_H_6_OS	114.2	1462.4	1154.845	1.10301
	103	Dimethyl sulfide	C75183	C_2_H_6_S	62.1	795.9	227.340	0.95470
Aromatic hydrocarbons	104	Ethylbenzene	C100414	C_8_H_10_	106.2	1137.6	541.182	1.09189

The substance suffixes M and D represent monomers and dimers of the same substance, respectively.

#### 3.1.2 GC-IMS fingerprint analysis

**FIGURE 3:**
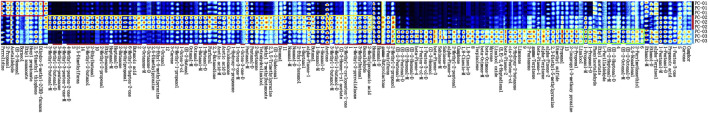
Fingerprint of VOCs in three parts of the *Poria cocos* samples. PC-01: Poria; PC-02: Poriae cutis; PC-03: Poria cum radius pini.

To further distinguish the VOCs present in various sections of *Poria cocos*, a comprehensive fingerprint analysis was performed on all VOCs, and the results are shown in Figure 3. In this figure, every row depicts the signal peaks derived from an individual sample, while each column represents the peaks of the identical volatile organic compound observed across multiple samples. The intensity of the color in each cell reflects the compound’s relative concentration, with more vivid hues indicating higher levels. Overall, the GC-IMS fingerprint profiles deliver a detailed summary of the volatile compounds in each sample and facilitate a visual evaluation of the differences among them. Further analysis revealed pronounced disparities in the volatile components among Poria (PC-01), Poriae cutis (PC-02), and Poria cum radice pini (PC-03), with PC-01 exhibiting relatively high contents of E-2-Nonenal, Borneol, 2-Propanol, Acetaldehyde, Ethyl nonanoate, Hexyl propionate, Dihydro-5-methyl-2(3H)-furanone, Pyrrolidine, 2,5-Dimethyl thiophene, and related compounds. PC-02 exhibited relatively high contents of Butanoic acid, 2-Methylpropanoic acid, Acetic acid, 3-Methyl-2-butenal, (E)-2-Butenal, 2-Methyl-2-propenal, Benzaldehyde, Furfural, Decanal, Nonanal, Octanal, Heptanal, Hexanal, 2-Ethylbutanal, Pentanal, 3-Methylbutanal, Butanal, 2-Methylpropanal, 3-Methyl-2-cyclopenten-1-one, 6-Methyl-5-hepten-2-one, 3-Octanone, 1-Hydroxy-2-propanone, Cyclohexanone, 1-Octen-3-one, 2-Heptanone, 4-Methyl-3-penten-2-one, 3-Methyl-2-pentanone, 2,3-Butanedione, 2-Butanone, Tetrahydrolinalool, 1-Octen-3-ol, 1-Octanol, 2-Ethyl-1-hexanol, 1-Heptanol, 1-Hexanol, 3-Methyl-1-butanol, 1-Butanol, 3-Methyl-2-butanol, 2-Methyl-1-propanol, γ-Butyrolactone, 2-Pentylfuran, 2,5-Dimethylfuran, 2-Ethyl-5-methylpyrazine, 2,3,5-Trimethylpyrazine, 2,6-Dimethylpyrazine, 3-Carene, Ethylbenzene, 1-Methyl-2-pyrrolidinone, and related compounds. PC-03 exhibited relatively high contents of Propanoic acid, L-Perillaldehyde, Phenylacetaldehyde, 2,6-Nonadienal, (E,E)-2,4-Heptadienal, (E)-2-Octenal, (E)-2-Heptenal, (E)-2-Hexenal, 2-Methyl-2-pentenal, (E)-2-Pentenal, 2-Propenal, Propanal, α-Terpineol, Linalool, (Z)-3-Hexenol, 1-Pentanol, 1-Penten-3-ol, 2-Methyl-2-propanol, 1-Propanol, Ethanol, Carvone, 3-Hydroxy-2-butanone, 1-Penten-3-one, 2-Pentanone, Ethyl acetate, Isoterpinene, γ-Terpinene, β-Ocimene, Limonene, α-Terpinene, Myrcene, Sabinene, β-Pinene, Camphene, α-Pinene, 1,8-Cineole, Camphor, Linalool oxide, 2-Isopropyl-3-methoxypyrazine, 2,3-Diethyl-5-methylpyrazine, Dimethyl sulfide, and related compounds.

### 3.2 Chemometrics

#### 3.2.1 Principal component analysis (PCA)

**FIGURE 4:**
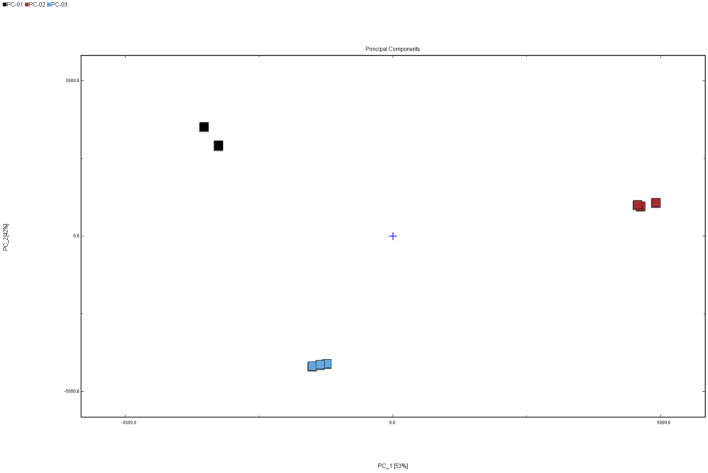
PCA score plot of VOCs in three parts of the *Poria cocos* samples. PC-01: Poria; PC-02: Poriae cutis; PC-03: Poria cum radius pini.

Principal Component Analysis (PCA) is an unsupervised multivariate statistical technique that effectively captures the underlying structure of original data. To visualize the differences in VOCs among different parts of *Poria cocos*, PCA was performed on the VOCs detected in the three sample groups: PC-01, PC-02, and PC-03. The results are presented in Figure 4. The cumulative contribution rate of the selected principal components reached 95%, with PC1 and PC2 accounting for 53% and 42% of the total variance, respectively. This indicates that the PCA model serves as a reliable discrimination model, and the chosen principal components play a dominant role in representing the relationships among the VOCs from different parts of *P. cocos*. In the PCA score plot, clear separation distances were observed among the samples PC-01, PC-02, and PC-03, demonstrating significant differences in the VOCs of Poria, Poriae cutis, and Poria cum radice pini.

#### 3.2.2 Cluster analysis (CA)

**FIGURE 5:**
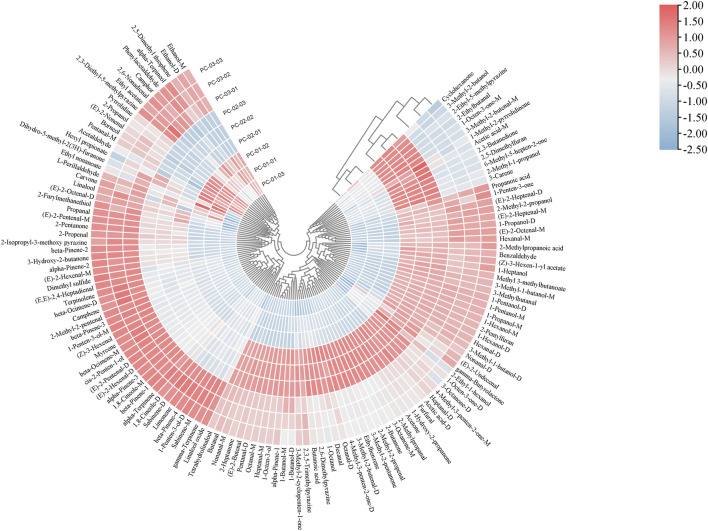
Cluster analysis heatmap of three parts of the *Poria cocos* samples. PC-01: Poria; PC-02: Poriae cutis; PC-03: Poria cum radius pini.

Cluster analysis (CA) is a multivariate analytical technique that is widely employed in the fingerprint analysis of group samples. As a non-parametric data interpretation method, it is straightforward to use and enables the visualization of complex datasets. A heatmap intuitively represents differences between groups through color gradients, where the depth of color corresponds to the concentration of substances—darker shades of red indicate upregulation (higher concentration), while darker shades of blue indicate downregulation (lower concentration). The peak data of volatile components from the three sample groups, PC-01, PC-02, and PC-03, were subjected to cluster analysis using TBtools software, with the results presented in Figure 5. The analysis revealed that the relative content of specific compounds was notably higher in each group. PC-01 was characterized by elevated levels of compounds such as Borneol, Dihydro-5-methyl-2(3H)-furanone, Ethyl nonanoate, (E)-2-Nonenal, Hexyl propionate, Pyrrolidine, Pentanal-M, 2-Propanol, and Acetaldehyde. There is a distinct set of compounds, including 1-Methyl-2-pyrrolidinone, Butanoic acid, 1-Octanol, 3-Methyl-2-cyclopenten-1-one, 2-Ethyl-1-hexanol, Decanal, Acetic acid-M, 1-Octen-3-ol, 2,3,5-Trimethylpyrazine, 2-Ethyl-5-methylpyrazine, Tetrahydrolinalool, Nonanal-M, 6-Methyl-5-hepten-2-one, 2,6-Dimethylpyrazine, 1-Octen-3-one-M, 1-Octen-3-one-D, Octanal-M, Octanal-D, Cyclohexanone, 3-Octanone-M, 3-Octanone-D, 3-Methyl-2-butenal-M, 3-Methyl-2-butenal-D, Heptanal-M, Heptanal-D, 2-Heptanone, 1-Butanol-M, 1-Butanol-D, 4-Methyl-3-penten-2-one-M, 4-Methyl-3-penten-2-one-D, 3-Carene, Ethylbenzene, 2-Methyl-1-propanol, 3-Methyl-2-butanol, (E)-2-Butenal, α-Pinene-1, 3-Methyl-2-pentanone, 2-Ethylbutanal, Pentanal-D, 2,3-Butanedione, 2,5-Dimethylfuran, 2-Butanone, 2-Methyl-2-propenal, Butanal, Acetone, and 2-Methylpropanal, showed higher abundance in PC-02. Conversely, PC-03 was enriched with compounds like L-Perillaldehyde, Carvone, α-Terpineol, Phenylacetaldehyde, 2,6-Nonadienal, Camphor, (E,E)-2,4-Heptadienal, 2,3-Diethyl-5-methylpyrazine, Linalool oxide, 2-Furylmethanethiol, (E)-2-Octenal-D, 2-Isopropyl-3-methoxypyrazine, (Z)-3-Hexenol, cis-2-Penten-1-ol, 3-Hydroxy-2-butanone, Terpinolene, γ-Terpinene, (E)-2-Hexenal-M, (E)-2-Hexenal-D, β-Ocimene-M, β-Ocimene-D, 1,8-Cineole-D, Limonene, 1-Penten-3-ol-M, Myrcene, 1-Penten-3-ol-D, 2-Methyl-2-pentenal, (E)-2-Pentenal-M, (E)-2-Pentenal-D, Sabinene-M, Sabinene-D, β-Pinene-2, β-Pinene-4, Camphene, α-Pinene-2, α-Pinene-3, 2-Pentanone, Ethyl acetate, 2-Isopropyl-3-methoxypyrazine, Propanal, Dimethyl sulfide, and Linalool. The results indicate that, while PC-03 and PC-02 exhibit a certain degree of clustering, the overall volatile component profiles among the three groups (PC-01, PC-02, and PC-03) are significantly distinct. This finding is consistent with and mutually validates the results obtained from the Principal Component Analysis (PCA).

#### 3.2.3 Euclidean distance

**FIGURE 6:**
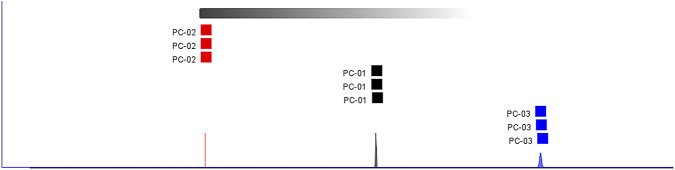
“Nearest-neighbor” fingerprint analysis of three parts of the *Poria cocos* samples. PC-01: Poria; PC-02: Poriae cutis; PC-03: Poria cum radius pini.

Euclidean distance, like Principal Component Analysis (PCA), serves as a clustering analysis method, representing the actual linear distance between two points in multidimensional space and reflecting the degree of similarity between the studied objects. A larger distance coefficient indicates a greater dissimilarity, showing a positive correlation with compositional differences. Evaluation of the different *Poria cocos* samples, as shown in Figure 6, reveals that the distance coefficients between PC-01 and PC-02, and between PC-01 and PC-03, were identical, indicating a consistent level of similarity between these pairs. In contrast, the largest distance coefficient was observed between PC-02 and PC-03, signifying the lowest degree of similarity. Consequently, the volatile components in the different parts of *P. cocos* exhibit significant differences.

#### 3.2.4 Partial least-squares discriminant analysis (PLS-DA)

**FIGURE 7:**
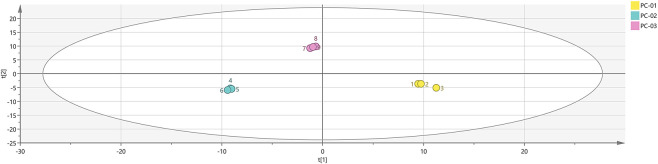
PLS−DA analysis of VOCs in 3 groups of *Poria cocos* samples. PC-01: Poria; PC-02: Poriae cutis; PC-03: Poria cum radius pini.

**FIGURE 8:**
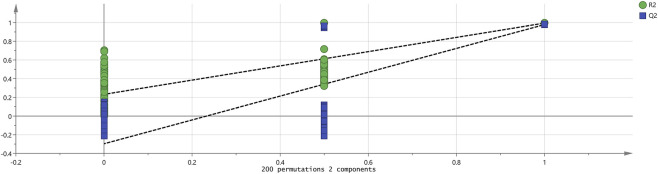
Permutation test results of VOCs in 3 groups of the *Poria cocos* samples.

Partial Least-Squares Discriminant Analysis (PLS-DA) is a supervised discriminant analysis statistical method distinct from PCA; it establishes a relationship between the characteristic attributes of samples and their classification targets to interpret observations, predict corresponding variables, and enhance classification accuracy. In this experiment, a PLS-DA model was constructed using SIMCA software by importing data from three sample groups, with the results presented in Figure 7. *R* ^2^ and Q^2^ values greater than 0.5 indicate an acceptable model fit, while values closer to 1 reflect stronger predictive capability. The PLS-DA score plot demonstrated a stable and reliable model with excellent predictive performance (R^2^X = 0.932, R^2^Y = 0.996, Q^2^ = 0.992). The proximity of *R* ^2^ and Q^2^ to 1 signifies a high goodness of fit. The considerable distances between the clusters for PC-01, PC-02, and PC-03 in the score plot reveal significant differences in volatile components among the different parts of Poria cocos, consistent with the conclusions drawn from the PCA. Additionally, the Variable Importance in Projection (VIP) score quantifies the contribution of each volatile compound to sample discrimination. A higher VIP value denotes a greater significance of the variable in differentiating groups, and variables with VIP >1 are generally considered influential (Supplementary Figure S2). The compounds with VIP>1 (i.e., identified as potential key differential markers) are as follows: (E)-2-Hexenal-D, (E)-2-Hexenal-M, (E)-2-Pentenal-D, (E)-2-Pentenal-M, 1-Penten-3-ol-D, 1-Penten-3-ol-M, β-Pinene-3, (Z)-3-Hexenol, 1,8-Cineole-M, α-Pinene-3, 2-Methyl-2-pentenal, Sabinene-M, cis-2-Penten-1-ol, β-Pinene-1, Dimethyl sulfide, Limonene, Myrcene, (E,E)-2,4-Heptadienal, 1,8-Cineole-D, 2,3-Diethyl-5-methylpyrazine, 3-Hydroxy-2-butanone, α-Pinene-2, β-Ocimene-M, β-Pinene-2, β-Pinene-4, γ-Terpinene, Terpinolene, (E)-2-Heptenal-D, (E)-2-Heptenal-M, (E)-2-Octenal-M, 2-Isopropyl-3-methoxypyrazine, 2-Pentanone, 2-Propenal, 3-Methyl-2-butanol, α-Terpinene, β-Ocimene-D, Camphene, Ethanol-D, Ethyl acetate, Linalool oxide, Phenylacetaldehyde, Propanal, Sabinene-D, 1-Octen-3-one-M, 1-Pentanol-D, 2,5-Dimethylfuran, 2-Ethyl-5-methylpyrazine, 2-Ethylbutanal, 6-Methyl-5-hepten-2-one, Hexanal-M, Ethanol-M, 1-Pentanol-D, 2,3-Butanedione, 2-Butanone, 2-Methyl-1-propanol, 2-Methyl-2-propanol, 2-Methyl-2-propenal, 3-Carene, 3-Methyl-2-butenal-D, 3-Methyl-2-pentanone, 4-Methyl-3-penten-2-one-D, and Ethylbenzene. To assess the potential overfitting of the model, a permutation test with 200 iterations was performed, with the results presented in Figure 8. The large slope of the regression line in the permutation test plot, along with the intercepts of *R* ^2^ = 0.252 and Q^2^ = −0.305, indicates the reliability of the constructed PLS-DA model. The negative value of Q^2^ confirms the robustness of the model and the absence of overfitting.

#### 3.2.5 Common and unique compound analysis

**FIGURE 9:**
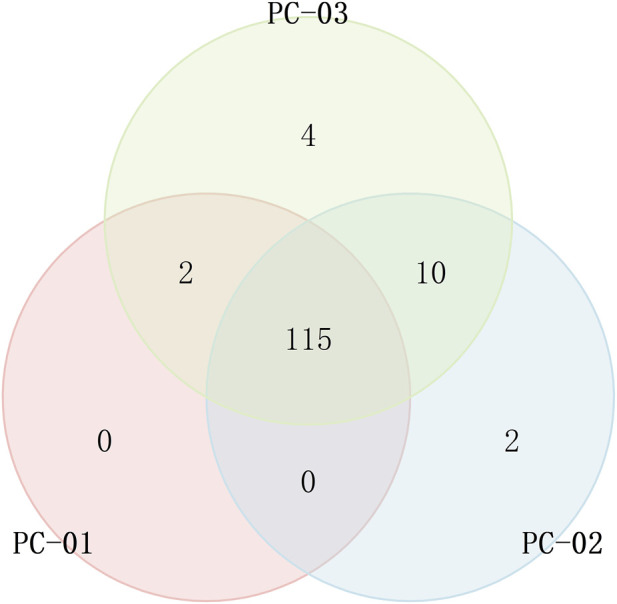
Venn diagram of aroma components of VOCs in three groups of samples. PC-01: Poria; PC-02: Poriae cutis; PC-03: Poria cum radius pini.

As shown in Figure 9, the Venn diagram showed the common and unique compounds, PC-01 and PC-03 contain two compounds that PC-02 lacks, Borneol and Phenylacetaldehyde. PC-02 and PC-03 contain ten compounds that PC-01 lacks, namely, (E, E)-2,4-Heptadienal, Linalool oxide, 2,3,5-Trimethylpyrazine, (E)-2-Octenal, Octanal, (E)-2-Hexenal, (E)-2-Pentenal, 1-Penten-3-one, Methyl 3-methylbutanoate, and Linalool. Moreover, two compounds, 3-Octanone and 4-Methyl-3-penten-2-one, were unique to PC-02, while four compounds, β-Ocimene, 1,8-Cineole, 2-Methyl-2-pentenal, and Sabinene, were exclusive to PC-03. The common and unique components identified by the Venn diagram align well with the PCA and CA results, as well as the fingerprint spectrum, further confirming the differences in VOCs between the three sample groups.

## 4 Discussion

The GC–IMS analysis (including three-dimensional spectra, two-dimensional topographic plots, and differential color spectra) visually demonstrates distinct differences in the volatile organic compounds among Poria, Poriae cutis, and Poria cum radice pini samples. Furthermore, fingerprint analysis of the volatile components reveals that sample PC-01 exhibits relatively high levels of Borneol, a compound with documented neuroprotective, anti-inflammatory, and antioxidant properties (Wang D. et al., 2025). In addition, this sample contains elevated levels of Ethyl nonanoate, a compound known to impart fruity and floral aroma notes to Poria (Wang X. et al., 2025). The sample PC-02 exhibited elevated concentrations of flavor compounds, including the aldehydes heptanal, octanal, nonanal, and decanal (Liu et al., 2025), as well as 1-octanol (Zong et al., 2025), tetrahydrolinalool, and 2,5-dimethylfuran. In contrast, sample PC-03 displayed a distinct volatile profile characterized by a high abundance of compounds with significant biological activities. Notably, major constituents in PC-03 were terpenoids and their derivatives—such as α-terpineol (Vuko et al., 2025), linalool, carvone, γ-terpinene, limonene, β-pinene (Tkaczenko et al., 2025), α-pinene, 1,8-cineole, and linalool oxide—which possess documented antioxidant and anti-inflammatory properties. Additionally, L-perillaldehyde, a compound recognized for its neuroregulatory functions (Ito et al., 2011), was detected at elevated levels. The volatile profile of PC-03 was further enriched by aldehydes that enhance flavor, including phenylacetaldehyde (Cheng et al., 2025), 2,6-nonadienal, (E,E)-2,4-heptadienal, (E)-2-octenal, (E)-2-heptenal, (E)-2-hexenal, 2-methyl-2-pentenal, (E)-2-pentenal, 2-propenal, and propanal, collectively contributing to the sample’s distinctive sensory attributes. These findings underscore the potential of PC-03 as a rich source of bioactive and flavor-active compounds, thereby supporting its prospective applications in functional food and phytopharmaceutical development.

Analyses employing principal component analysis (PCA), Cluster Analysis (CA), and Euclidean distance metrics revealed distinct differences among the anatomical sections. These findings indicate that the volatile component profiles of *Poria cocos* exhibit significant variation across its different parts.

Partial Least-Squares Discriminant Analysis (PLS-DA) revealed significant differences in the volatile component profiles among the three sample groups, thereby reinforcing previous conclusions. This finding not only substantiates the distinct chemical compositions of the various parts of Poria cocos but also establishes a robust theoretical foundation and practical rationale for expanding the medicinal applications of its anatomical sections, particularly Poriae cutis and Poria cum radice pini. Employing PLS-DA, this study further identified the following volatile organic compounds as primary differential markers: (E)-2-Hexenal-D, and Ethylbenzene. Studies have revealed that both Poriae cutis and Poria cum radice pini contain notably high levels of aldehydes, which are characterized by their diverse aromatic profiles, including fruity, floral, and occasionally distinct almond or nut-like notes, making them valuable in fragrance and flavor applications. Notably, furfural, identified in Poriae cutis, has demonstrated significant antibacterial and anti-inflammatory activities, as evidenced by research on citrus-derived analogues (Sánchez-Recillas et al., 2017). Additionally, decanal, which is present in these parts, exhibits potential in mitigating exogenous skin aging (Kang et al., 2020).

The α-terpineol present in Poria cum radice pini exhibits analgesic properties that can mitigate cancer-related pain (Gouveia et al., 2018), while linalool demonstrates potent antioxidant and anti-inflammatory activities capable of delaying cellular aging (Azırak and Özgöçmen, 2023). In addition, linalool possesses anxiolytic, antitumor, sedative, and hypnotic properties (Koriem and El-Qady, 2023).

This study employed gas chromatography–ion mobility spectrometry (GC–IMS) in conjunction with chemometric methods to systematically investigate the compositional differences in volatile organic compounds (VOCs) among distinct medicinal parts of Poria cocos—namely, Poria, Poriae cutis, and Poria cum radice pini. The volatile components were comprehensively profiled, leading to the identification of part-specific volatile markers. The results revealed significant disparities in the VOC profiles across the different parts, with distinct distribution patterns of key bioactive constituents demonstrating pronounced part-specificity. These findings provide a compositional basis for the targeted dietary or medicinal application of specific Poria cocos parts and lay the groundwork for establishing a part-specific quality evaluation system. Furthermore, this research offers theoretical guidance for the differentiated development of Poria cocos in functional foods, pharmaceutical products, and other high-value applications, thereby promoting resource-efficient utilization. Overall, the study underscores the importance of a multi-component, comprehensive approach in traditional Chinese medicine and advances the modernized innovation of herbal resources through the application of advanced analytical technologies.

## 5 Conclusion

This study employed Gas Chromatography–Ion Mobility Spectrometry (GC-IMS) coupled with multivariate chemometric techniques—including Principal Component Analysis (PCA), Cluster Analysis (CA), and Partial Least-Squares Discriminant Analysis (PLS-DA)—to systematically characterize the volatile compositional profiles of various medicinal parts of *P. cocos* (Poria, Poriae cutis, and Poria cum radice pini). A non-targeted analysis identified 104 volatile organic compounds (VOCs), comprising 31 aldehydes, 28 alcohols, 7 terpenoids, 15 ketones, 8 heterocyclic compounds, 5 esters, 4 carboxylic acids, and 6 hydrocarbons.

Chemometric analysis revealed significant variations in the profiles of volatile components among the different medicinal parts of *P. cocos*. Specifically, Poriae cutis was characterized by an enrichment of compounds such as butanoic acid, 2-methylpropanoic acid, acetic acid, 3-methyl-2-butenal, (E)-2-butenal, 2-methyl-2-propenal, benzaldehyde, furfural, decanal, nonanal, octanal, heptanal, hexanal, 2-ethylbutanal, and pentanal. In contrast, Poria cum radice pini exhibited a markedly high abundance of propanoic acid, L-perillaldehyde, phenylacetaldehyde, (E)-2-heptenal, (E)-2-hexenal, 2-methyl-2-pentenal, (E)-2-pentenal, 2-propenal, and propanal. Additionally, the Poria component contained elevated levels of volatile substances, including (E)-2-nonenal, borneol, 2-propanol, acetaldehyde, ethyl nonanoate, and hexyl propionate. Among them, 3-Octanone and 4-Methyl-3-penten-2-one were unique to PC-02, β-Ocimene, 1,8-Cineole, 2-Methyl-2-pentenal, and Sabinene, were exclusive to PC-03.

This study establishes a foundation for the precise consumption and therapeutic application of various parts of *P. cocos*. It specifically explored the material basis underpinning the calming effects of *P. cocos*, identifying compounds such as L-Perillaldehyde, which is associated with neural regulation, and linalool, recognized for its anti-anxiety, anti-tumor, sedative, and hypnotic properties. These findings are expected to advance the development and utilization of different parts of *P. cocos* in health product innovation.

## Data Availability

The original contributions presented in the study are included in the article/Supplementary Material, further inquiries can be directed to the corresponding authors.
