# Supplementary figures and images for: Comparative analysis of volatile organic compounds in different parts of Poria cocos
Source: Front Chem. 2026 Apr 1;14:1777381. doi: 10.3389/fchem.2026.1777381 (PMC13079666; doi:10.3389/fchem.2026.1777381)

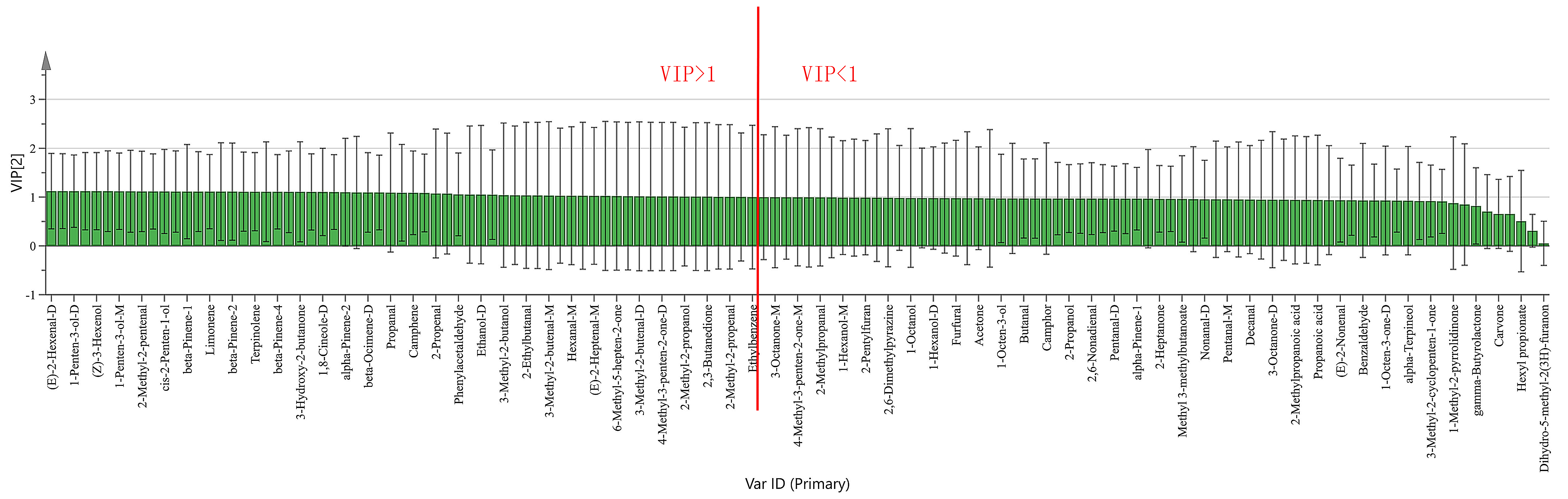

Supplement: Supplementary file 1 [file Image2.png]

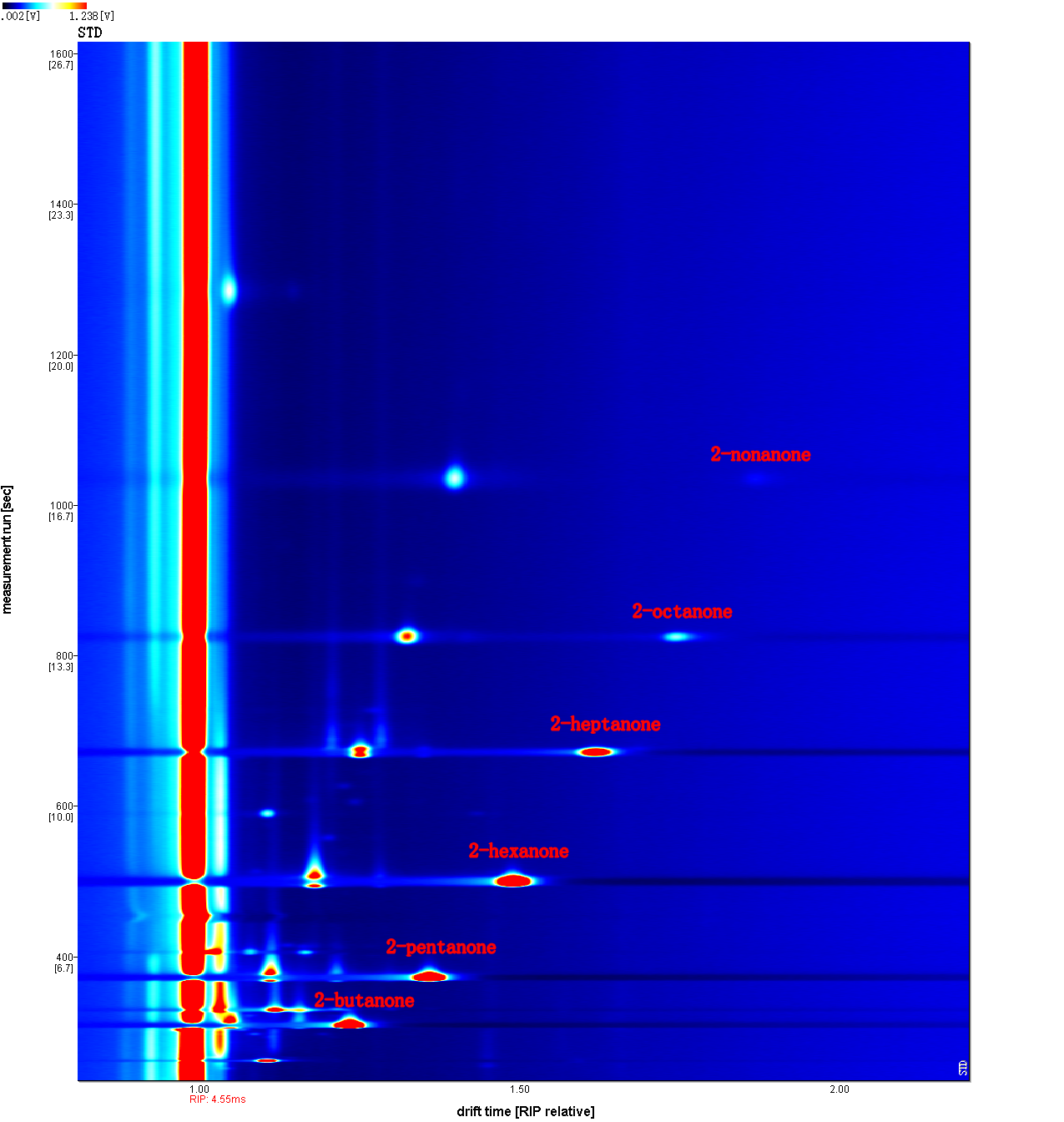

Supplement: Supplementary file 3 [file Image1.png]
